# Assessing Maternal Capabilities in the SHINE Trial: Highlighting a Hidden Link in the Causal Pathway to Child Health

**DOI:** 10.1093/cid/civ851

**Published:** 2015-11-11

**Authors:** Cynthia R. Matare, Mduduzi N. N. Mbuya, Gretel Pelto, Katherine L. Dickin, Rebecca J. Stoltzfus

**Affiliations:** 1 Division of Nutritional Sciences, Cornell University, Ithaca, New York; 2 Zvitambo Institute for Maternal and Child Health Research, Harare, Zimbabwe; 3 Department of International Health, Johns Hopkins Bloomberg School of Public Health, Baltimore, Maryland

**Keywords:** caregiver capabilities, child caregiving, intervention design, impact evaluation

## Abstract

A potential bottleneck for increasing the adoption of child health interventions has been limited attention to designing actions that are built on the essential role that caregivers play in determining their effectiveness. In the Sanitation Hygiene Infant Nutrition Efficacy (SHINE) trial, we utilize the concept of maternal capabilities to examine participants' skills and attributes that affect their ability to provide appropriate care for their young child, fully engage with trial interventions, and influence the response to these interventions at the household level. We hypothesize that the impact of SHINE interventions on child stunting and anemia will be modified by these maternal capabilities. Drawing upon multiple theories, we identify and define critical maternal capabilities domains, and describe how they are measured in the trial. Description of maternal capabilities and their role as potential modifiers on impact will increase understanding of the impact of SHINE interventions, and the generalizability of our findings.

The health, nutrition, and other developmental opportunities afforded to a child from conception until the age of 2 years are critically important to their immediate and long-term well-being [1]. Undernutrition continues to be a public health problem in developing countries, and is a leading cause of disability and death in young children [1]. Among preschool-aged children, 165 million children are stunted [2], close to 300 million are anemic [3], >50 million are wasted [4], and 45% of all deaths in this age group can be attributed to these and other forms of undernutrition [2]. Zimbabwe, like other developing countries, has a high burden of undernutrition among preschool-aged children; about 10% have a low birth weight, a third have stunted growth, and more than half (56%) are anemic [5]. In Zimbabwe, child feeding practices in the first 2 years are suboptimal: 5.8% of children were exclusively breastfed for 6 months (despite of high breastfeeding initiation and continuation), and 8.4% of children between 6 and 23 months of age received a minimal acceptable diet (an indicator that combines both dietary diversity and meal frequency) [6].

Although a great deal is known about what needs to be done to improve the nutrition of women and young children [7], and political will to address this is issue is growing (for example, 55 countries have committed to the Scaling Up Nutrition movement) [8], progress in tackling malnutrition has been slow, evidenced by the fact that only 1 country (Colombia) is on track to meet the nutrition targets set by the World Health Assembly [9]. One reason for this could be the lack of consideration for the important role that behavioral factors play in ensuring the healthy growth for infants and young children, specifically the preventive, disease management, and healthcare-seeking behaviors performed by their caregivers.

The basic conceptual framework linking caregivers at the household level to nutrition outcomes was effectively articulated >25 years ago. In this framework, care was defined as the “provision in the household and the community of time, attention, and support to meet the physical, mental, and social needs of the growing child and other household members” [10], or more simply, the practices that “translate food security and healthcare resources into a child's well-being” [11]. In many contexts, the mother is the primary caregiver, and therefore has a central role in determining the infant's health and nutritional outcomes. In some cases, others (eg, fathers and grandparents) are also primary caregivers, but in more cases, they act as secondary caregivers, assisting the mother or sharing the role of caregiver.

The Sanitation, Hygiene, and Infant Nutrition Efficacy (SHINE) trial in Zimbabwe is implementing 2 packages of interventions, one targeting infant and young child feeding (IYCF) and the other targeting water, sanitation, and hygiene (WASH) behaviors, with the primary goal of reducing stunting and anemia at 18 months of age. These SHINE interventions are in line with 3 of the 13 interventions that have been identified as being feasible and cost-effective and fall under the broader category of promoting good nutritional practices [7]. The behaviors promoted through the SHINE trial interventions are particularly aimed at minimizing the ingestion of fecal matter by and improving the hygienic care of infants and young children, thereby reducing the risk for diarrheal and other infectious diseases. All of these behaviors also require *deliberate* action to be taken by a caregiver, if they are to impact the health and growth of the child. In view of this, it is essential to understand caregivers and their capabilities as the most proximal participants in the delivery of these interventions. In doing so, researchers will be better equipped to develop programs that facilitate the adoption of and compliance with appropriate behaviors for child health. As such, the SHINE trial program impact pathway (PIP) considers a specific set of maternal attributes, which we have broadly named “caregiver capabilities.” An exploration of caregiver capabilities is the focus of this article, as we view them as important effect modifiers in the SHINE PIP [12].

## THE CAREGIVER CAPABILITIES FRAMEWORK

The caregiver capabilities framework builds on work by several theorists, notably Engle et al [13], who extended the 1990 UNICEF (United Nations Children's Fund) framework to define several resources a caregiver would need to provide appropriate care, and Nussbaum, whose conceptualization of the human capabilities approach [14] included a defined list of capabilities [15]. These resources for care and human capabilities are summarized in Table 1. Marrying these concepts, we have adopted the term “caregiver capabilities” to broadly represent the skills and attributes of a caregiver that determine their ability to care for a young child in ways that produce positive nutrition, health, and development outcomes (Caregiver Capabilities Working Group, Cornell). This characterization of caregiver capabilities leads to the proposition that child health and nutrition interventions will have limited utility if caregiver capabilities are low and not addressed by the intervention. Therefore, child health and nutrition outcomes can be improved by increasing the resources that caregivers have access to, or by improving caregiver capabilities, or a combination of both.

**Table 1.** List of Resources for Care and the Human Capability Set

Resources for Care	Human Capability Set
Education, knowledge, and beliefs	Life
Health and good nutritional status	Bodily health
Mental health/lack of stress	Bodily integrity
Autonomy, control of resources, and intrahousehold allocation	Senses, imagination, and thought
Workloads and time availability	Emotions
Social support from family members and community	Practical reason
Control of resources and intra-household allocation	Affiliation
Self-efficacy	Other species
	Play
	Control over one's environment

In the context of the SHINE trial, all caregivers enrolled in the study and from whom data are being collected are mothers. In acknowledgement of this fact, in this article we refer to caregiver capabilities specifically as maternal capabilities. The SHINE trial selected a subset of maternal capabilities as the focus of attention. These are: mental health (depressive symptoms), stress, perceived physical health, autonomy, perceived social support, mothering self-efficacy, time use, and perceived time stress. From this list, we define positive maternal capabilities as “having good physical health,” “low levels of depressive symptoms,” “less stress,” “more social support,” “high levels of mothering self-efficacy,” and “being more autonomous.”

Drawing from multiple sources of theory and empirical research, these indicators are defined in Table 2 and brief summary justifications follow.

**Table 2.** Definitions of Constructs Comprising Caregiver Capabilities

Construct	Definition
Perceived physical health	Perceptions of one's physical well-being
Mental health [16]	A state of well-being in which an individual can realize their abilities, cope with the normal stresses of life, work productively and fruitfully, and is able to make a contribution to their community
Stress [17]	The emotional experience triggered by events or other stimuli and accompanied by specific biochemical, physiological, and behavioral changes
Perceived social support [18]	Perceived exchanges of physical and psychosocial resources provided through a process of interaction in relationships, which leads to improved coping, esteem, belonging, and competence
Decision-making autonomy [19]	The capacity to manipulate one's environment through control over resources and information to make decisions about one's own concerns or about close family members
Gender norm attitudes [20]	Traditional or egalitarian beliefs about male and female gender norms
Time use	The allocation of time to different activities
Perceived time stress	The caregiver's perceptions of the adequacy of the time they have to attend to their different roles
Mothering self-efficacy [21]	The degree to which parents perceive themselves as capable and effective in the parenting role

### Physical Health

Women in developing countries face many physical health challenges, from multiple sources, including high burdens of diseases (eg, tuberculosis, human immunodeficiency virus/AIDS, and iron-deficiency anemia, are 3 of the largest contributors of disability-adjusted life-years lost among women of childbearing age in these contexts [22]). In considering physical health, we distinguished between objective and subjective measures. It is widely acknowledged that people may perceive themselves to be healthy despite suffering from a disease, and alternately, others will perceive themselves to have poor health when there is no objective indication of disease [23]. For the purposes of constructing maternal capabilities in the SHINE trial, perceived physical health will be considered.

### Mental Health and Stress

Although many aspects of mental illness can affect caregiving, we focus on depression. It is the most prevalent mental health disorder globally and accounts for about 10% of all years lived with disability [24]. Depression often presents with depressed mood, loss of interest or pleasure, decreased energy, feelings of guilt or low self-worth, disturbed sleep or appetite, and poor concentration [25]. Depression affects a mother's ability to appropriately care for her child, her capacity to positively interact with the baby [26], and her ability to seek healthcare resources [27]. Furthermore, its incidence is about 3 times higher in the year following birth, compared to other periods in a woman's life [28], and this coincides with the time that infants are especially vulnerable, in need of “others” to care for them and sensitive to any insults to their health and nutritional well-being. Consequently, postnatal depressive symptoms can have detrimental effects on the health and development of a baby [27, 29]. The high vulnerability of women in developing countries to depression in the postnatal period may be associated with hormonal changes [29], compounded by poverty, lower social status for women, alcoholism, domestic violence, and lack of autonomy [30].

### Perceived Social Support

Social support during the postnatal period, especially from partner and other household members, is widely recognized as essential for the well-being of both mother and baby [31, 32]. Social support has also been highlighted in ecological models of health behavior, including Bronfenbrenner's ecological systems theory [33]. We focus on assessing women's functional social support, for which 4 elements have been described: (1) informational, (2) instrumental, (3) emotional, and (4) companionship support [34]. To date, much of the analysis of the role of social support and child care practices in developing countries has focused on breastfeeding, and on the provision of informational and emotional support through peer counselors [35, 36].

### Autonomy

Autonomy is a multidimensional construct that has been defined by Dixon [37] as the extent to which a woman has access to and control over material and social resources within her household, her community, and in society at large [37]. Autonomy is often defined in 5 dimensions (emotional, physical, economic, knowledge, and decision making) [38] and, considering the provisioning of care for children in the cultural context of Zimbabwe, we chose to focus on household decision-making autonomy. Related to autonomy, we also assess women's attitudes towards gender norms, a broader indication of the environment in which women exercise autonomy, or not. Recently, it has been posited that women with high levels of autonomy might experience a loss of identity if this autonomy appears to be a violation of gender norms in the household and/or communities they reside [39, 40].

### Workload and Perceived Time Stress

Although reasonable workloads and the availability of adequate time for women are essential for the provision of care, they are the least explored construct among those identified in the extended UNICEF framework [13]. Many women in rural parts of developing countries average 18-hour days, and it has been recognized that these women often have very little, if any, free or leisure time [41]. Many of these women may be involved in formal and informal income-earning activities, but their roles nearly always include domestic activities, including unpaid subsistence farming, collecting water, gathering fuel (eg, firewood), cooking, other household chores, and activities related to child care, which are often invisible [42]. As their time has been described as a finite and zero-sum condition [13], it inevitably influences their engagement with nutrition interventions and programs. In one study aimed at improving infant feeding practices in rural China, more than half of the mothers who reported not practicing the recommended behaviors gave time constraints as their reason for not doing so [43].

### Mothering Self-efficacy

Self-efficacy, or a construct similar to it, is common to many of the health behavior theories used to explain, predict, and change health behaviors, including Bandura's social cognitive theory [44], the health belief model [45], and the theories of reasoned action and planned behavior [46, 47]. In many situations, it appears to be a proximal determinant of behavior. Mothering self-efficacy reflects women's sense of self in their role as mothers, and in turn, how confident and competent they are in that role [21]. Although much of the research on the relationship between self-efficacy and infant and young child nutrition behaviors has focused specifically on breastfeeding practices [48–51], other work has shown that low levels of mothering self-efficacy can influence other aspects of childcare [52]. Bandura notes that mothers who have higher levels of mothering self-efficacy are more likely to express interest in and commitment to their role [53]. Ruchala and James proposed high levels of mothering self-efficacy as the most important predictor of positive mothering behaviors [54].

## ASSESSMENT OF MATERNAL CAPABILITIES

With the exception of stress, time use, and perceived time stress, caregiver capabilities will be assessed using survey items adapted from various instruments. The instruments from which these items were adapted are outlined in Table 3. No appropriate instruments to assess time use and time stress were found in the literature. Therefore, we used qualitative methods to develop items specifically for the SHINE trial. For stress, saliva samples will be collected to test for levels of cortisol, a method that is used widely [60].

**Table 3.** Assessment of Caregiver Capabilities Constructs

Construct	Survey Items Adapted From^a^	Baseline	Months Postpartum			
			1	6	12	18
Perceived physical health	RAND 36-Item Health Survey [55]	✓		✓		✓
Depression	Edinburgh Postnatal Depression Scale [56]	✓	✓	✓	✓	✓
Stress	Salivary cortisol^a^	✓				
Perceived social support	Interpersonal support evaluation list [34] Medical Outcomes Study Social Support Survey [57]	✓		✓		✓
Decision-making autonomy	Demographic and Health Survey	✓		✓		✓
Gender norm attitudes	Gender Norm Attitudes Scale [20]	✓		✓		✓
Time use	No instrument found in the literature; items were developed for the SHINE trial	✓		✓		✓
Perceived time stress	No instrument found in the literature; items were developed for the SHINE trial	✓		✓		✓
Mothering self-efficacy	Parenting Sense of Competence Scale [58] Parenting Self-agency Measure [59]	✓		✓		✓

^a^ With the exception of salivary cortisol, a biochemical test for stress.

Following translation of the items into 2 local languages, Ndebele and Shona, all instruments used to assess caregiver capabilities underwent cognitive testing to ensure validity of the questions and response options. Cognitive testing involves in-depth interviewing method to evaluate potential sources of error in survey questions [61]. We used the “think-aloud” and verbal probing techniques concurrently to assess the following basic processes involved in responding to survey questions: comprehension of questions (is the respondent's perception of the question in line the intent of the question?); retrieval from memory information relevant to the questions (what thought processes does the respondent go through to come up with a response for the question?); decision process (is the question sensitive or likely to invoke a socially desirable response?); and response process (are the response options appropriate and/or adequate?) [61]. Additionally, the final survey instruments were pilot-tested. Both the cognitive testing and pilot testing were conducted in a district adjacent to the SHINE study area prior to data collection. Table 3 highlights the time points at which each of the variables will be assessed, starting at baseline.

The number of items in the scales we developed or adapted to assess the different caregiver capabilities ranges from 5 to 14. For each of these multi-item scales, composite scores will be generated. For further analyses related to other trial components and discussed in Mbuya et al [12], these scores will be used as continuous scales or as categorical scales. Results from initial psychometric testing, based on data from about 2000 women enrolled in the SHINE Trial, show that the internal consistency and reliability of the scales are acceptable, with Cronbach α ranging from 0.70–0.88. This provides solid evidence that, in addition to being theoretically sound, the items used to assess each of the constructs comprising caregiver capabilities are indeed measuring the same underlying construct.

## SHINE TRIAL HYPOTHESES RELATED TO CAREGIVER CAPABILITIES

The behaviors of caregivers of young children are clearly an important step in the pathway to child health, and the SHINE PIP [12] makes explicit that the final delivery of the interventions by caregivers is an aspect of the fidelity of implementation of our interventions. We view caregiver capabilities as an effect modifier of intervention impact, potentially shaping the heterogeneity of the effects of the interventions. We hypothesize that mothers with strong capabilities will be able to utilize resources provided through the SHINE trial (WASH hardware, education, and nutritional supplements) to achieve greater increases in child health outcomes. In other words, stronger caregiver capabilities will strengthen the association between maternal knowledge and WASH and IYCF behaviors. The role of caregiver capabilities likely pertains to multiple other caregiving behaviors, such as optimal breastfeeding practices, participation in vaccination programs, and appropriate care of the sick infant, that are essential for the prevention and treatment of infectious diseases in infants and are synergistic with SHINE interventions.

Additionally, we hypothesize that household visits by village health workers will have a positive effect on some of the caregiver capabilities, particularly time stress, mental health, social support, and mothering self-efficacy. This has been reported previously in an evaluation of a food assistance program in rural Haiti, which had a strong behavior change communication component delivered by community-level workers [62]. In the Haitian evaluation, the investigators found a positive impact of program participation on measures of participants' self-rated health, mental stress, and time stress [62]. However, because SHINE interventions are adding more activities to caregivers' daily routines, it is possible that the interventions will increase their time stress.

## CONCLUSIONS

It has been noted previously that understanding the characteristics that promote or constrain the uptake and effective utilization of health interventions could result in more efficient and effective use of resources available for child health promotion [63]. Therefore, building on a solid base of theory, we propose to study the effects of maternal capabilities on a multipronged intervention to improve care and feeding of infants and young children. We designed a holistic and longitudinal quantitative survey assessment of maternal capabilities to be used in the SHINE trial, with specific hypotheses about how these might mediate or modify the intervention effects. It is our hope that the analysis of maternal capabilities in the SHINE trial will reveal avenues for their further exploration in the context of other nutrition and child health interventions, and in the context of other societies.
